# Expression of CD1d by astrocytes corresponds with relative activity in multiple sclerosis lesions

**DOI:** 10.1111/bpa.12733

**Published:** 2019-06-06

**Authors:** Fraser G.W. Muir, Zahra Samadi‐Bahrami, George R. Wayne Moore, Jacqueline A. Quandt

**Affiliations:** ^1^ Department of Pathology & Laboratory Medicine, International Collaboration on Repair Discoveries (ICORD) University of British Columbia Vancouver Canada; ^2^ Department of Pathology & Laboratory Medicine, Department of Medicine (Neurology), International Collaboration on Repair Discoveries (ICORD) University of British Columbia Vancouver Canada; ^3^ Department of Pathology & Laboratory Medicine University of British Columbia Vancouver Canada

**Keywords:** antigen presentation, CD1d, demyelination, lipid, multiple sclerosis, white matter

## Abstract

The CD1 protein family present lipid antigens to the immune system. CD1d has been observed in the CNS of MS patients, yet no studies have quantitatively characterized this expression and related it to inflammatory demyelinative activity in MS plaques. In this study, we set out to localize and quantify the presence of CD1d expression by astrocytes in MS brain tissue lesions. Formalin‐fixed, paraffin‐embedded MS and control brain tissues were examined. Lesions were classified as active, chronic active or chronic silent. Using immunofluorescence, the density of CD1d‐positive cells was determined in active lesions, chronic active lesion edges and chronic active lesion centers. The percentage of CD1d‐positive cells that were GFAP‐positive was also determined in each of these regions. CD1d immunoreactivity was significantly increased in MS compared to control tissue, was significantly more prevalent in areas of active demyelination, and colocalized with GFAP‐positive reactive astrocytes. Increases of CD1d immunoreactivity in the CNS of MS patients being greatest in areas of active demyelination and localized to GFAP‐positive astrocytes lend support to the hypothesis of a lipid‐targeted autoimmune process contributing to the pathogenesis of MS.

## Introduction

The pathological hallmarks of multiple sclerosis (MS) plaques are inflammation, demyelination and gliosis; remyelination and axonal loss are also recognized as important components of the lesion 17. MS has long been thought to be an autoimmune disorder directed against some as yet undetermined epitope in the central nervous system (CNS), particularly the myelin sheath 16, 25. Numerous studies have investigated various protein constituents of the myelin sheath as putative autoimmune antigens but to date no convincing protein candidate has been found. Very little attention has been directed to lipid autoimmunity in the pathogenesis of MS. Lipids are presented to the immune system by a family of MHC‐like molecules known as CD1 23. CD1 molecules are grouped into class 1, composed of CD1a‐c and class 2, which consists of solely CD1d 7. CD1 molecules have previously been described in the CNS of MS patients. An increase in the expression of CD1b was found in MS lesions, which was restricted primarily to areas of active demyelination, with minimal reactivity found elsewhere, and in control white matter 4. CD1b was expressed primarily in hypertrophic astrocytes and could be seen in perivascular inflammatory cells both within the lesion and surrounding it. CD1a expression was found expressed in the parenchyma with CD1a^+^ dendritic cells found in the perivascular space of two early active lesions and a chronic active lesion 22. In lesions from a single case of acute MS, CD1d was localized to reactive astrocytes and occasional microglia 9.

CD1d expression in MS may be relevant to the disease process, as CD1d‐restricted T cells have been implicated in pathogenesis in mouse models of MS; the best characterized of these are the natural killer T (NKT) cells 5. In experimental autoimmune encephalomyelitis (EAE) the prototypical autoimmune model of MS, activated NKT cells were found to be protective, and their activation and protective function was lost in CD1d knockout mice 15, 24. In wild‐type animals, these activated NKT cells were capable of infiltrating the CNS of EAE mice and promoted a shift to a more suppressive T helper (Th) 2 response, and inhibited proinflammatory Th1 and Th17 responses 11, 24.

In MS patients, the overall numbers of circulating invariant NKT (iNKT) cells are reduced compared to healthy controls. However, the CD4^+^ iNKT cell population is relatively spared 1. These CD4^+^ iNKT cells release IL‐4 and are skewed to a Th2 profile, which suggests they may contribute to disease modulation, similar to what is observed in murine models of MS 1. This decrease in iNKT cells is also present in the CNS of MS patients, with very few iNKT cells observed in lesions 10. γδ T cells are another subset of T cells capable of recognizing a wide range of antigens including lipids and glycosphingolipids in the context of CD1d 8, 14. It has been shown that large portions of all T cells in acute lesions of MS patients are of the γδ type, and that these were primarily constrained to the leading edge of chronic active lesions 21, 26.

Given these data, we hypothesized that a lipid‐specific autoimmune response may contribute to MS disease pathogenesis, and the characterization of CD1d quantitatively in different types of MS lesions would shed light on its relevance to the disease process.

## Materials and Methods

### Patient selection

This study was approved by the Clinical Research Ethics Board of the University of British Columbia (H01‐70430). The study was conducted on archival, paraffin‐embedded, formalin fixed brain tissues obtained from 11 MS cases, and four normal control cases which exhibited no other neurodegenerative condition (see Tables 1 and 2 for patient and control data). The diagnosis of MS was confirmed by a neuropathologist and then anonymized to MS(X) or normal case/NC(X) designations for sectioning and staining/processing.

**Table 1:** MS patient information.

MS case	Age (years)	Disease duration	PMI (hours)	MS type	# Blocks/# lesions	Anatomical location	Lesion type		
							Active	Chronic active	Classification per Kuhlmann *et al* 13
1	35/M	20 years	17	RPMS	1/1	Left frontal lobe	1		nd
2	44/F	> 15 years	48	PPMS	1/1	Left frontal lobe		1	Mixed active/inactive and demyelinating
3	32/F	nd	<24	MS	1/1	Left middle frontal gyrus		1	Mixed active/inactive and demyelinating
4	53/F	nd	nd	MS	1/2	Right posterior parietal lobe		2	Mixed active/inactive and demyelinating
5	57/F	>13	nd	MS	1/1	Left parietal lobe	1		Active and early demyelinating
6	40/M	<1	nd	MS	1/1	Right posterior frontal lobe	1		Active and early demyelinating
7	54/F	14 years	96	MS	1/1	Left frontal lobe		1	Mixed active/inactive and demyelinating
8	48/F	16 years	nd	MS	1/4	Right frontal lobe	2	2	Chronic active lesions: mixed active/inactive and demyelinating
									Active lesions: 1 active and demyelinating; 1 nd.
9	49/F	11 years	48	MS	1/1	Right inferior temporal lobe		1	Mixed active/inactive and demyelinating
10	45/F	>25 years	nd	MS	1/2	Right frontal lobe		2	Mixed active/inactive and demyelinating
11	58/F	30 years	72	MS	1/1	Left hippocampus and para‐hippocampal gyrus		1	Mixed active/inactive and demyelinating

Post‐mortem interval (PMI) was time between time of death and autopsy. Clinical records from referred cases were incomplete in some cases and did not allow for the classification of the multiple sclerosis (MS) or a determination of the post‐mortem interval.

Abbreviations: F = female; M = male; nd = no data; PPMS = primary progressive MS; RPMS = relapsing‐progressive MS.

**Table 2:** Control patient information.

Normal Case	Age (years)	PMI (hours)	Cause of death	Number of blocks	Anatomical location
1	54/M	72	Pulmonary emboli, metastatic adeno carcinoma	1	Right superior and middle temporal gyri
2	71/F	72	Myocardial infarct, atherosclerosis	1	Right rostral cingulate gyrus
3	71/F	72	Cardiac arrhythmia	2	Left rostral middle frontal gyrus
					Right inferior parietal lobe
4	54/F	48	Bronchopneumonia, renal failure, portal vein thrombosis	1	Right rostral cingulate gyrus

Post‐mortem interval (PMI) was time between time of death and autopsy.

Abbreviations: F = female; M = male.

### Tissue processing and immunohistochemistry

Luxol fast blue (LFB) staining was conducted according to protocols described previously 12 on five micron‐thick sections of archival, formalin‐fixed paraffin‐embedded tissue and immunohistochemistry was done using anti‐HLA‐DR (Dako, Glostrup, Denmark), and myelin basic protein (MBP)(Dako) utilizing an avidin‐biotin immunoperoxidase kit with Nova Red as the chromogen (Vector Laboratories, Burlingame, CA). Most of this study was completed prior to the new classification system proposed by Kuhlmann *et al* 13. Subsequently, where tissue was still available, this classification system was applied to lesions in this study, utilizing additional immunohistochemical staining for myelin oligodendrocyte glycoprotein (MOG) (R&D Systems, Minneapolis, MN, USA) and CD68 (Dako). Immunofluorescent staining was performed on serial sections from white‐matter regions of the selected blocks, and human lymph node as a positive control for CD1d and ionized calcium‐binding adapter molecule‐1 (Iba‐1, a marker of macrophage/microglia) using a multi‐step staining protocol for CD1d (AbD Serotec, Hercules, CA, USA), Iba‐1 (AbCam, San Francisco, CA, USA), and GFAP for astrocytes (Dako, Glostrup, Denmark). All stains used donkey anti‐mouse AlexaFluor 647, donkey anti‐goat AlexaFluor 568 and donkey anti‐rabbit AlexaFluor 488 as secondaries (ThermoFisher/Molecular Probes, Eugene, OR, USA) to stain CD1d, Iba‐1, and GFAP, respectively. Nuclear staining was done using 4′,6‐Diamidino‐2‐Phenylindole (DAPI, ThermoFisher/Molecular Probes, Eugene, OR), and this was followed by staining with 0.15% Sudan Black B (SBB) in 50% ethanol (Fisher Scientific, Fair Lawn, NJ, USA) in order to minimize autofluorescence, adapted from past papers 3, 19, 20. To investigate the specificity of SBB to stain myelin immunofluorescence was done against MBP. All negative control slides were stained using the appropriate isotype control antibodies at matched concentrations. For full information on antibodies used for immunohistochemistry and immunofluorescence, their concentrations, and their isotype controls see Supplementary Tables S1 and S2.

### Lesion classification

**Figure 1:**
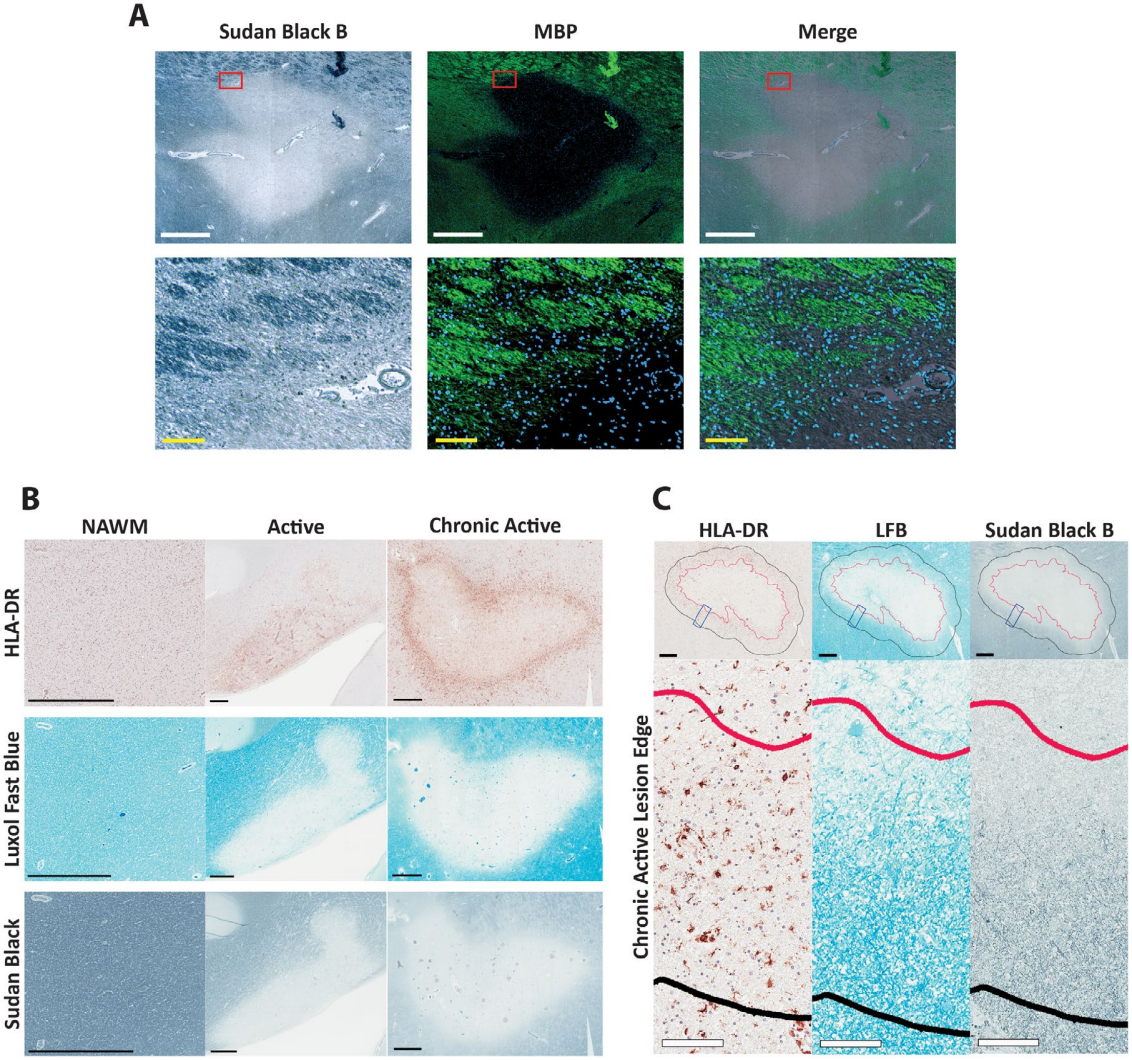
*Classification of MS lesions based on histochemical and immunohistochemical staining characteristics*. **A.** Brightfield of a MS lesion showing Sudan Black B staining with corresponding fields imaged in immunofluorescence showing MBP positivity in green. An overlay of both Sudan Black B and MBP is shown as a merge. The bottom row of images are magnified views of the regions outlined in red in the upper row of images. White scale bars = 1000 µm, yellow scale bars = 100 µm. **B.** Lesions were classified as either active or chronic active, based on the presence or absence of myelin (LFB staining) and reactive microglia/macrophages (HLA‐DR). Chronic active lesions lacked myelin (LFB staining) throughout the center, with HLA‐DR positivity around the periphery of the lesion. Active lesions had variable amounts of myelin throughout while also having HLA‐DR positivity throughout the lesion; normal‐appearing white matter (NAWM) exhibited no loss of myelin, with limited HLA‐DR positivity. Sudan Black B staining for myelin showing the same pattern as observed for LFB. (The chronic active lesions were all classified as mixed active/inactive and demyelinating by the system proposed by Kuhlmann *et al* 13; active lesions, where tissue was available, were all active and early demyelinating by this system). Scale bar = 1mm. **C.** Defining lesion edge and histologic characteristics of lesion edge. In chronic active lesions, an active edge of 500 µm (black line) was defined extending beyond the edge of complete demyelination (red line), defined by Sudan Black B positivity as seen in **A** and **B**. This edge was characterized by an increase in HLA‐DR staining density, as well as positive myelin staining (LFB). Black scale bar = 500 µm, white scale bars = 100 µm. Insets are higher magnifications of the regions outlined by blue rectangles in the upper row.

Lesion classification was done using characteristics previously established based upon the presence, absence and/or distribution of both LFB (myelin), as well as HLA‐DR (microglia and macrophages) 6, 18. Chronic active lesions were characterized by a prominent ring of HLA‐DR positivity (Figure 1), with definitively less positivity in the center, and a loss of LFB positivity within the lesion. Active lesions showed uniform HLA‐DR positivity throughout, with variable retention of LFB positivity—evidence of an early ongoing, inflammatory demyelinating response. Normal‐appearing white matter (NAWM) was defined as mild HLA‐DR positivity with full retention of LFB staining. Where tissue was available these lesions were also stained with MBP, MOG and CD68 to enable classification as active, mixed active/inactive and inactive with or without ongoing demyelination 13. Active demyelinating lesions were further characterized into early and late demyelinating lesions based on the presence of major and small molecular weight myelin proteins (MOG) vs. presence of major myelin proteins (MBP), respectively 13. Based on this classification, we found that chronic active lesions examined were indeed mixed active/inactive and demyelinating with MBP or MOG rarely or not detected in macrophages. This was different than three of five active lesions that could be re‐examined which were designated active and early demyelinating for the presence of both MBP and MOG in macrophages within these lesions (Table 1, Supplementary Table S3).

### Image acquisition and analyses

The entire tissue section was imaged in fluorescence using the 20x objective with 2x2 binning in ZEN 2.0 (Carl Zeiss GmbH) on a Zeiss Axio Observer Z1. Autofocus was run every third tile, using GFAP as the reference channel, and DAPI as a reference channel in the negative controls. All fluorescent images were stitched using DAPI as the reference channel. Brightfield images of LFB and HLA‐DR slides were acquired using an Aperio CS2 Digital Pathology Scanner (Leica Biosystems) using the 20× objective.

Lesions were first outlined using the acquired brightfield images. This was done by outlining the extent of complete demyelination based on the absence of SBB staining. The lesion edge in chronic active lesions was defined as the region between the demyelinated and myelinated white matter interface, and a line outlined 500 micrometers outside this first line. Both outlines were obtained on the brightfield images of SBB staining and were transferred to the immunofluorescent images in ZEN 2.0.

Thresholding for each fluorescent channel was performed using values obtained from positive control tissues. The threshold for CD1d and Iba‐1 was the level at which positive cells were clearly distinguished with minimal background in the control lymph node tissue containing clearly positive and negative populations; this was performed for GFAP in brain tissue. Negative controls were analyzed to control against autofluorescence. Cell counting was performed using a 100 by 100 micrometer grid overlaid on the images using Zen 2.0 and using an unbiased counting frame. Quantification was performed in a systematic random manner. From the randomized starting location, every third grid square was then quantified, in both the horizontal and vertical axes, such that 1/9^th^ of the entire lesion area was quantified (see Supplementary Figure S1 for details). In the sampled grid squares, the number of nuclei were counted, followed by assessing which nuclei were associated with CD1d‐positivity. Next, the number of these CD1d‐positive cells that were co‐labeled with anti‐GFAP or anti‐Iba‐1 was determined. Cell density results were expressed as cells/mm^2^.

### Statistics

In all analyses, each lesion was treated as a separate n, with no pooling between lesions taken from the same patient or other patients. Data were compiled in Microsoft Excel, then transferred to Prism 6 (GraphPad Software, Inc) for statistical analyses and graphing. When comparing more than two groups Kruskal‐Wallis test with Dunn's multiple comparison test was used. The Mann‐Whitney test was used to identify differences between two groups while Wilcoxon matched‐pairs rank test was used for paired measures. In all cases, a p value of less than 0.05 was considered significant, and reported results used two‐tailed analyses. In analyses where a percentage of cells was calculated, results with no positive cells were not included since a percentage of no value cannot be calculated.

## Results

### Lesion characterization

MS cases analyzed in this study averaged in age at 46 ± 8.6 years (range 32–58 years) with an average disease duration of MS diagnosis of 16.1 ± 8.3 years (range 1–30 years, Table 1). Tissues from these nine females and two males were from frontal, temporal and parietal lobe regions with one from the hippocampus. Five NAWM regions were identified (1 each from 3 different blocks/cases and another 2 from a single block/case) and examined in this study. Tissues from normal cases aged 54–71 years of age were from similar brain regions (Table 2). Our samples did not contain active grey matter lesions/cortical lesions and therefore these could not be included in our analyses.

SBB which was used to minimize autofluorescence in the tissue also stains myelin lipids; this is illustrated by similar staining patterns of multiple regions with SBB and MBP in Figure 1A. For chronic active lesions, the area outlined by the extent of complete demyelination—as evidenced by lack of SBB staining—was considered the lesion center, while as noted in the Materials and Methods, the lesion edge was defined as an area extending 500 µm out from the interface between myelinated and demyelinated white matter (Figure 1C). The outlining based on SBB corresponded well with where demyelinated white matter abutted myelinated white matter as shown by LFB positivity; this area also corresponded with the rim of increased HLA‐DR positive cell staining which typically declined beyond this lesion edge (Figure 1B,C).

### CD1d‐positive cell density is increased in MS lesions

**Figure 2:**
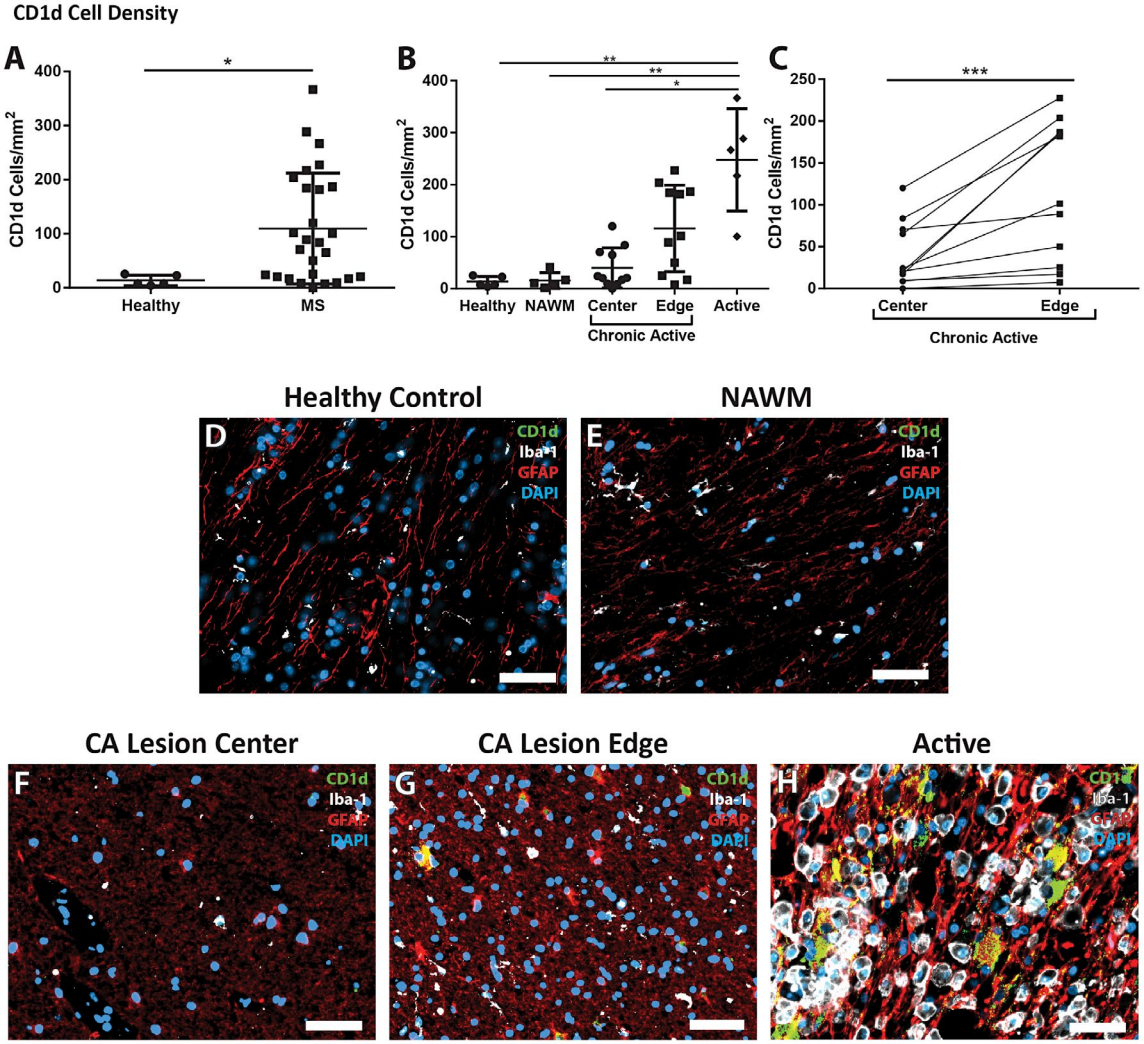
*Changes in the density of CD1d in MS patients.* **A.** Multiple sclerosis tissue showed a significant increase in density of CD1d‐positive cells when compared to controls (*n* = 27, and 5, respectively). **B.** The increase in CD1d‐postive cell density was significantly different in different lesion types, and different areas within those lesions. Active lesions had a much greater density than chronic active lesion centers, NAWM, and controls (*n* = 5, 11, 5 and 5, respectively). The edges of chronic active lesions had a higher density of CD1d‐positive cells than controls and NAWM, though did not achieve significance (*n* = 11, 5 and 5, respectively). **C.** Density of CD1d was significantly greater in the chronic active lesion edge than in the paired chronic active lesion center (***P = 0.001, *n* = 11 per group, Wilcoxon matched‐pairs signed‐rank test). Immunofluorescence shows highly reactive astrocytes (red, GFAP), but not microglia (white, Iba‐1), immunoreactive for large amounts of CD1d (green) in the active lesion (**H**) with double‐labeling appearing as yellow, while no CD1d is evident in the control (**D**) or NAWM (**E**). The chronic active lesion center (**F**) and chronic active lesion edge (**G**) demonstrate a more intermediate astrocyte phenotype (less hyperplastic and more fibrillary), though there are appreciable levels of CD1d in the reactive astrocytes within the chronic active lesion edge. DAPI (blue) as nuclear stain; scale bars = 50 µm. *P ≤ 0.05, **P ≤ 0.01. Bars represent the mean, and error bars the standard deviation. (**A**, **C**) Mann‐Whitney, (**B**) Kruskal‐Wallis with Dunn's multiple comparison test.

When comparing the CD1d‐positive cell density of all MS tissue against control tissue, the density of CD1d‐positive cells was significantly increased in MS compared to healthy tissues (109.5 ± 102.3 cells/mm^2^ vs. 13.70 ± 9.776 cells/mm^2^, respectively, *P* = 0.0184) (Figure 2A). The density of CD1d‐positive cells in NAWM was not significantly different than healthy control tissues at 15.89 ± 15.4 cells/mm^2^. When the MS tissue region subtypes were compared against each other, and to control tissue, active lesions were found to have a significantly higher density of CD1d‐positive cells than healthy control tissue (247.9 ± 98.43 cells/mm^2^ vs. 13.70 ± 9.776 cells/mm^2^, *P* = 0.0057) and chronic active lesion centers (247.9 ± 98.43 cells/mm^2^ vs. 40.10 ± 38.59 cells/mm^2^, *P* = 0.0248). The density of CD1d‐positive cells in chronic active lesion edge also tended to be higher density than controls but did not reach significance (115.9 ± 83.24 cells/mm^2^ vs. 13.70 ± 9.776 cells/mm^2^, *P* = 0.1503) (Figure 2B).

Paired analyses of chronic‐active lesion centers to their chronic‐active lesion edges in Figure 2C revealed that CD1d‐positive cell density was greater at the legion edge (115.9 ± 83.24 cells/mm^2^) than at the center of the lesion (40.10 ± 38.58 cells/mm^2^, *P* = 0.001). The higher density of CD1d‐positive cells at the legion edge or rim region, where HLA‐DR‐positivity is higher, associates higher CD1d positivity with regions of greater HLA‐DR‐positivity.

### Proportion of CD1d‐positive cells

**Figure 3:**
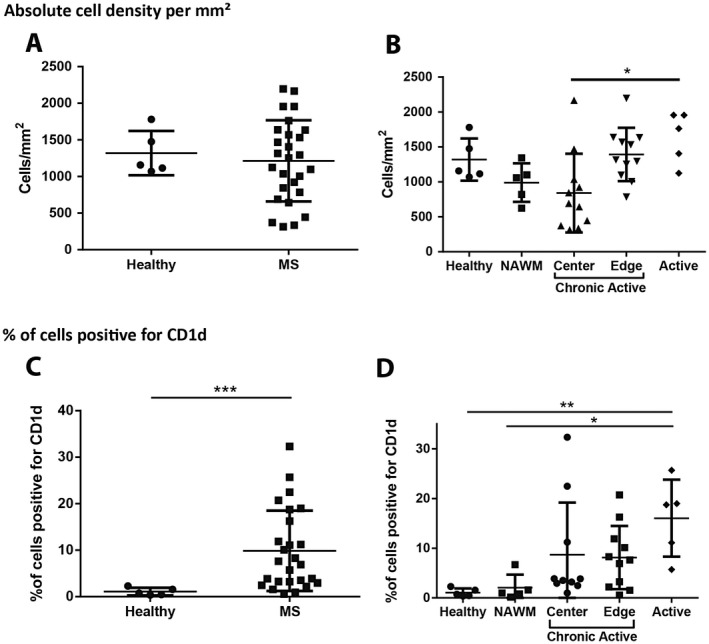
*Absolute cell density and % of CD1d cells in MS lesions.* **A.** Total cell density was not increased when all MS regions were compared against control samples (*n* = 27 and 5, respectively, Mann‐Whitney test). **B.** Active lesions showed a significant increase in the total cell density when compared against chronic active lesion centers (*n* = 5, and 11, respectively, Kruskal‐Wallis with Dunn's multiple comparison test). *P ≤ 0.05. Bars represent the mean, and error bars the standard deviation. **C.** A significantly higher proportion of the cells in MS tissues were positive for CD1d than in those of control samples (*n* = 26, and 5, respectively, Mann‐Whitney test). **D.** Although there was no significant difference between positivity in healthy controls and NAWM, there was a significant increase in the percentage of cells staining with CD1d in active MS lesions when compared to control (*n* = 5 control, 5 NAWM, 5 active, 10 chronic active lesion center, and 11 chronic active lesion edge samples, Kruskal‐Wallis with Dunn's multiple comparison test). **P ≤ 0.01, ***P ≤ 0.001. Bars represent the mean, and error bars the standard deviation.

The inflammatory process brings many infiltrating cells into the MS lesion and possibly also the surrounding area; there was a possibility that the increase in CD1d‐positive cell density shown in Figure 2B was simply due to hypercellularity in lesions and peri‐lesional areas. To exclude the possibility that the increased CD1d‐positive cell density was due to increased total cell density, the absolute cell density was calculated in MS, control, as well as in the NAWM and individual MS tissue regions (Figure 3A,B). There was no significant difference in the mean cell densities between MS and the control tissue (Figure 3A); when individual MS tissue regions were compared, the only region showing a difference in overall cell density was the group of active lesions when compared against the chronic active lesion center tissue (1641 ± 366.4 cells/mm^2^ vs. 839.7 ± 563.3 cells/mm^2^, respectively *P* = 0.0185) (Figure 3B).

Similar to CD1d‐positive cell density in brain tissue, there were increases in the percentage of cells staining positive for CD1d in MS tissue relative to control tissues (9.860 ± 8.655% vs. 1.078 ± 0.8249%, respectively, *P* = 0.0007) (Figure 3C). No significant difference was observed between NAWM at 2.048 ± 2.641% and healthy control tissues. Notably, the percent of cells showing CD1d immunoreactivity was higher in active lesions than in healthy controls (16.04 ± 7.750% vs. 1.078 ± 0.8249%., *P* = 0.0070) and NAWM (16.04 ± 7.750% vs. 2.048 ± 2.2641%., *P* = 0.0328) (Figure 3D).

### GFAP‐positive cells are the majority of CD1d^+^ cells in MS tissues

**Figure 4:**
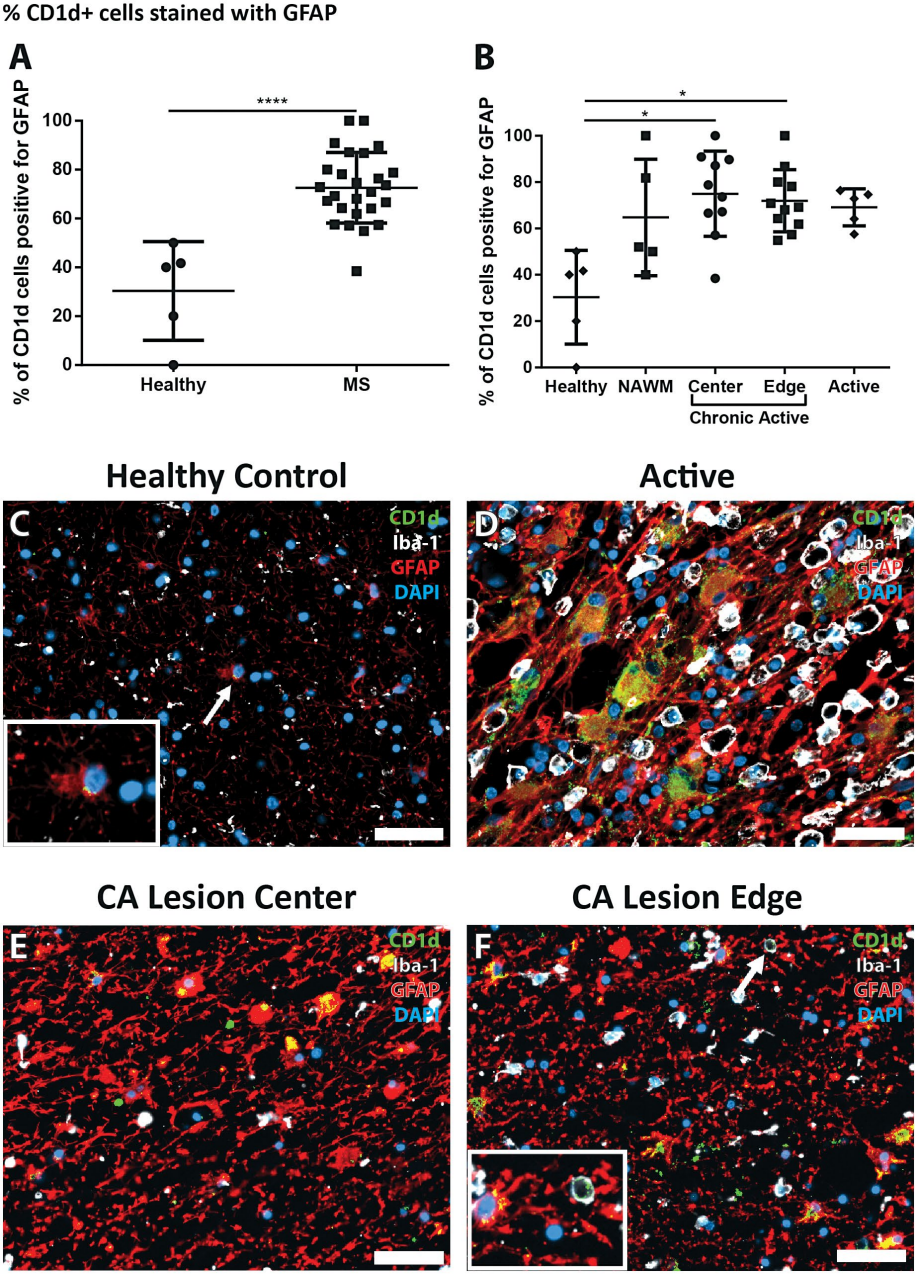
*Percentage of CD1d‐positive cells co‐labeling with GFAP is higher in MS lesion tissues.* **A.** In MS tissues CD1d‐positive cells were significantly more likely to be GFAP‐positive than in control tissue (*n* = 26, and 5, respectively, Mann‐Whitney test). **B.** The percentage of CD1d‐postive cells also positive for GFAP was significantly higher in chronic active lesion centers and chronic active lesion edges when compared to control tissue (*n* = 10, 11, and 5, respectively, Kruskal‐Wallis with Dunn's multiple comparison test). **C.** A rare CD1d‐positive astrocyte in control tissue showing minimal cytoplasmic positivity, with staining restricted to the perinuclear region (arrow, inset). (**D**, **E**, **F**) Large, reactive astrocytes in MS lesions showing cytoplasmic CD1d‐positivity are more common in the active lesion (**D**) than in the lesion edge (**F**) and both more common than in the lesion center (**E**). The occasional CD1d‐, Iba‐1‐positive cell was seen in MS tissues (**F**, arrow, inset). DAPI as nuclear stain, CD1d in green, GFAP in red, Iba‐1 in white; scale bars = 50 µm. *P ≤ 0.05, ****P ≤ 0.0001. Bars represent the mean, and error bars the standard deviation.

We next sought to establish the cell types that were expressing CD1d in healthy control tissues or MS lesions. Co‐staining for cell phenotype markers showed that the percentage of CD1d‐positive cells showing immunoreactivity for GFAP was significantly higher in MS than in the control tissue, (72.57 ± 14.45% CD1d^+^GFAP^+^ vs. 30.33 ± 20.22% CD1d^+^GFAP^+^, respectively; *P* ≤ 0.0001) (Figure 4A). There was no difference in the proportion of cells staining for GFAP in healthy control tissues compared to NAWM at 64.76 ± 25.13%. The proportion of CD1d^+^ cells which showed GFAP immunoreactivity in chronic‐active lesion edge and chronic‐active lesion centers was greater than observed in control healthy tissues (71.95 ± 13.41% and 74.97 ± 18.38% CD1d^+^GFAP^+^ vs. 30.33 ± 20.22% CD1d^+^GFAP^+^ (*P* = 0.0312, and *P* = 0.0112, respectively) (Figure 4B,C‐F).

In contrast, the % of CD1d^+^ cells which co‐stained with Iba‐1 antibodies was very low. There was no significant difference between the % of CD1d^+^/Iba1^+^ cells in healthy control tissue and NAWM (5.46 ± 12.20% and 1.82 ± 4.07%), nor were there increases in any type of lesion tissue obtained from MS patients (active at 7.52 ± 9.74%, chronic‐active lesion center at 3.09 ± 3.39% or chronic active lesion edge at 4.87 ± 4.68%).

### Morphology of cells showing dual immunoreactivity for GFAP and CD1d in MS lesions

Qualitatively we noted marked differences in cell morphology of CD1d‐positive cells between the different MS lesion types. We observed a pattern in the morphology with regions with higher HLA‐DR positivity (ie, active lesions, and chronic active lesion edges) showing CD1d‐positive cells that were much larger, hypertrophic, and had CD1d‐positivity throughout a larger portion of the cytoplasm (Figure 4D–F). These cells were typically astrocytes with a reactive, hypertrophic phenotype, increased GFAP content and cell size, and occasionally multiple nuclei.

## Discussion

The current study represents a comprehensive characterization of CD1d in MS lesions with specific quantification of its localization to GFAP^+^ astrocytes in different lesion types. CD1d‐positive cells that did not co‐label with GFAP were detected at low levels, and introduce the possibility that another cell type in the CNS may be expressing low levels of CD1d. Notably, CD1d immunoreactivity is confined primarily to regions of immune activity and mirrors the localization of CD1d‐restricted T cells described in other studies 21, 26. The finding of CD1d in MS lesions fits well with previous studies showing CD1b was often upregulated in active lesions, and that CD1d could be expressed in an acute MS lesion 4, 22. By establishing a well‐characterized method to distinguish different regions of MS lesions based on the presence or absence of myelin it was possible to quantify the presence of CD1d within areas of active demyelination (active lesions, and the edge of chronic active lesions), as well as in areas of complete demyelination (chronic active lesion centers). This regional analysis distinguishes our study as the first to establish that CD1d presence in MS lesions can vary significantly depending on the degree of immune activity and where it is localized in a lesion. Our study showed negligible increases in CD1d expression in regions of NAWM of tissues examined, suggesting that CD1d expression and relevant roles in immunity are less likely to precede active inflammation and associated immune responses. The presence of CD1d in the areas of active demyelination and lesion expansion in MS more readily implicates CD1d in these later immune processes. Previous studies showing distribution of γδ T cells greatest at the active periphery of MS lesions and accumulating in the early stages of MS lesion development, are similar to our finding for CD1d 26. This supports the likelihood that lipid antigen presentation to γδ T or lipid sequestration and co‐expression with CD1d^+^ cells may be occurring during the active phases of demyelination in MS lesions, which could have a very important role in the expansion of the lesion and a lengthened immune response. The lack of iNKT cells in MS lesions does not necessarily suggest that they are not effector cells in human MS, rather, this may simply reflect the challenge of capturing a population of effector cells that act over a very brief window of time in a disease as chronic as MS 10. From this one may infer a similar process to that in mouse models of MS, where NKT cells are capable of infiltrating the CNS and interact with lipid‐antigen presenting cells to exert some protective effect 11, 24. This study has shown that reactive astrocytes express CD1d in the CNS, and therein astrocytes may possess the potential to present lipids within the CNS of MS patients. This supports the previous findings from Battistini *et al* and Höftberger *et al* who found CD1b and CD1d expression primarily on reactive astrocytes 4, 9.

The presence of CD1d in the areas of active demyelination and lesion expansion, but not NAWM in MS, fits well with a potential role in pathways related to inflammatory damage. The distribution of γδ T cells primarily at the active periphery of MS lesions, and extending outwards into the surrounding white matter and accumulating in the early stages of MS lesion development 26 is very similar to what we observed for CD1d. This observation points to the likelihood of lipid antigen presentation to γδ T cells as an early event in active phases of demyelination, expansion of the lesion, as well as a driver of the ensuing immune response given the large quantities of IL‐17 γδ T cells can produce. Another hint at a pathogenic role for γδ T cells is their previously demonstrated ability to respond to sulfatide, a major component of myelin 2. In contrast, the lack of iNKT cells 10 in MS lesions, with their proposed ability to regulate this immune response, means the engagement of γδ T cells capable of propagating the immune response may tip the balance away from immunomodulation toward inflammation and tissue damage. Taken together, this study provides further rationale to consider the relevance of CD1d and associated lipid antigen presentation in the pathogenesis of MS.

## Conflict of Interest

The authors have no conflicts of interest to declare.

## Supporting information

**Figure S1** **.** Imaging and sampling workflow. **A.** The slide is first acquired in brightfield with the 10x objective. **B.** The extent of demyelination is outlined (yellow) based on the absence of Sudan Black B. **C.** These outlines are transferred to the 20x immunofluorescence image. **D.** A line (red) is drawn 500µm outside the extent of demyelination. **E.** A 100x100µm grid is laid over the image. **F.** The first grid square fitting fully within the outlined area is identified (yellow star). **G.** Two numbers between one and three are randomly generated. **H.** The numbers generated in G are used as grid references from the grid square identified in F to determine the starting grid for quantification. Every third grid square in the horizontal and vertical axes are then quantified, such that 1/9th of all grid squares are quantified. Yellow scale bar = 5000µm, red scale bar = 2000µm, white scale bar = 200µm.
**Table S1** **.** Antibodies used for immunohistochemistry.
**Table S2** **.** Antibodies used for immunofluorescence.
**Table S3** **.** Lesion classification scheme.
