# Supplementary figures and images for: Expression of CD1d by astrocytes corresponds with relative activity in multiple sclerosis lesions
Source: Brain Pathol. 2019 Jun 6;30(1):26–35. doi: 10.1111/bpa.12733 (PMC6916356; doi:10.1111/bpa.12733)

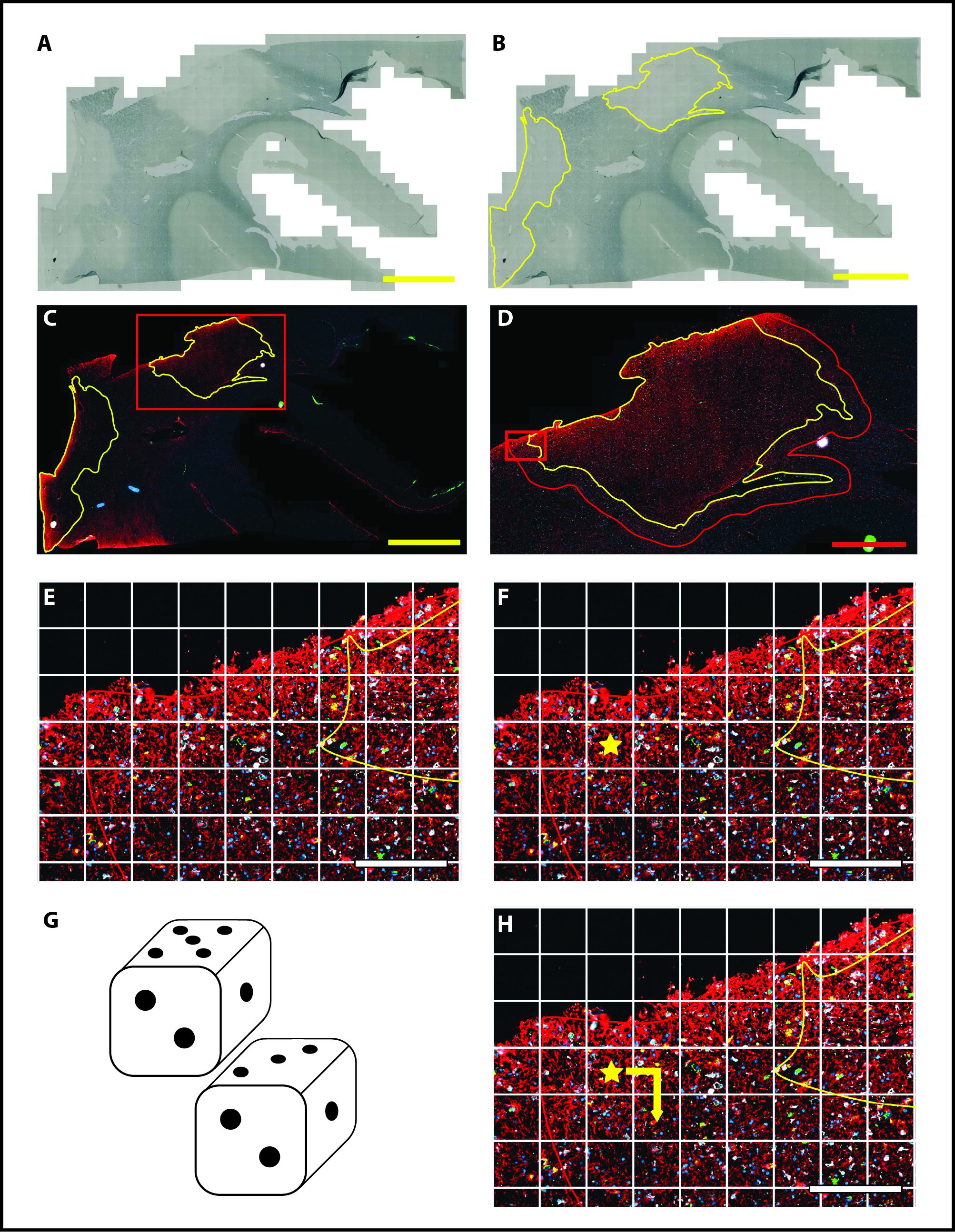

Supplement: Supplementary file 1 [file BPA-30-26-s003.tif]
