# Supplementary material for: Expression of CD1d by astrocytes corresponds with relative activity in multiple sclerosis lesions
Source: Brain Pathol. 2019 Jun 6;30(1):26–35. doi: 10.1111/bpa.12733 (PMC6916356; doi:10.1111/bpa.12733)
Supplement: Supplementary file 2 [file BPA-30-26-s002.docx]

## Supplementary Table 1

## Antibodies used for immunohistochemistry

| **Primary Antibody** | **Species/Isotype** | **Working Concentration** | **Manufacturer** | **Catalogue number** |
| --- | --- | --- | --- | --- |
| HLA-DR | Mouse IgG1 | 0.77μg/mL | Dako | M0775 |
| CD68 | Mouse IgG3k | 0.3µg/mL | Dako | M0876 |
| MBP | Rabbit | 0.0112g/L | Dako | A0623 |
| MOG | Goat | 0.2mg/mL | R&D Systems | AF2395 |
| **Normal IgG** | **Species/Isotype** | **Working Concentration** | **Manufacturer** | **Category number** |
| Mouse IgG |  | 0.77µg/mL (HLA-DR) | Sigma | 9269 |
|  |  | 0.3µg/mL (CD68) |  |  |
| Goat IgG |  | 0.2mg/mL | Sigma | I5256 |
| Rabbit IgG |  | 0.0112g/L | Dako | X0936 |
