# Supplementary material for: Expression of CD1d by astrocytes corresponds with relative activity in multiple sclerosis lesions
Source: Brain Pathol. 2019 Jun 6;30(1):26–35. doi: 10.1111/bpa.12733 (PMC6916356; doi:10.1111/bpa.12733)
Supplement: Supplementary file 3 [file BPA-30-26-s001.docx]

## Supplementary Table 2

## Antibodies used for immunofluorescence

| **Primary Antibody** | **Species/Isotype** | **Working Concentration** | **Manufacturer** | **Catalogue number** |
| --- | --- | --- | --- | --- |
| CD1d | Mouse IgG1 | 0.01mg/mL | AbD Serotec | MCA982G |
| Iba-1 | Goat | 0.004mg/mL | AbCam | Ab107159 |
| GFAP | Rabbit | 0.0058g/L | Dako | Z0334 |
| MBP | Rabbit | 0.014g/L | Dako | A0623 |
| CD1d | Rabbit | 0.001mg/mL | AbCam | Ab151768 |
|  | | | | |
| **Secondary Antibody** | **Wavelength (nm)** | **Working Concentration** | **Manufacturer** | **Catalogue number** |
| Donkey anti-mouse | 647 | 0.0067mg/mL | Molecular Probes | A21571 |
| Donkey anti-goat | 568 | 0.0067mg/mL | Molecular Probes | A11058 |
| Donkey anti-rabbit | 488 | 0.0067mg/mL | Molecular Probes | A21206 |
|  | | | |  |
| **Normal IgG** | **Working Concentration** | **Manufacturer** | **Catalogue number** |  |
| Mouse IgG | 0.01mg/mL | Sigma | 9269 |  |
| Goat IgG | 0.004mg/mL | Sigma | I5256 |  |
| Rabbit IgG | 0.0058g/L (GFAP) | Dako | X0936 |  |
|  | 0.014g/L (MBP) |  |  |  |
|  | 0.001mg/mL (CD1d) |  |  |  |
