# Supplementary material for: Expression of CD1d by astrocytes corresponds with relative activity in multiple sclerosis lesions
Source: Brain Pathol. 2019 Jun 6;30(1):26–35. doi: 10.1111/bpa.12733 (PMC6916356; doi:10.1111/bpa.12733)
Supplement: Supplementary file 4 — Figure S1 . Imaging and sampling workflow. A. The slide is first acquired in brightfield with the 10x objective. B. The extent of demyelination is outlined (yellow) based on the absence of Sudan Black B. C. These outlines are transferred to the 20x immunofluorescence image. D. A line (red) is drawn 500µm outside the extent of demyelination. E. A 100x100µm grid is laid over the image. F. The first grid square fitting fully within the outlined area is identified (yellow star). G. Two numbers between one and three are randomly generated. H. The numbers generated in G are used as grid references from the grid square identified in F to determine the starting grid for quantification. Every third grid square in the horizontal and vertical axes are then quantified, such that 1/9th of all grid squares are quantified. Yellow scale bar = 5000µm, red scale bar = 2000µm, white scale bar = 200µm. Table S1 . Antibodies used for immunohistochemistry. Table S2 . Antibodies used for immunofluorescence. Table S3 . Lesion classification scheme. [file BPA-30-26-s004.docx]

Supplementary Table 3

| **Anon case #** | **Block** | **Lesion (s) used in the block** | **Anatomical location** | **MBP in MƟ** | **MOG in MƟ** | **CD68/ HLA-DR/ Hypercell distribution** | **Hypercell Border** | **Degree Demy** | **Classification per Kuhlmann et al 2017** |
| --- | --- | --- | --- | --- | --- | --- | --- | --- | --- |
|  | | | | | | | | | |
| **Chronic Active Lesions** | | | | | | | | | |
| 2 | D | 1 | Left frontal lobe | Occ | Occ | Chronic Active | <25% (1+) | Few (1+) | Mixed active/ inactive and demyelinating |
| 3 | C | 1 | Left middle frontal gyrus | Rare | Neg | Chronic Active | <25% (1+) | Few (1+) | Mixed active/inactive and demyelinating |
| 4 | D | CA1 | Right posterior parietal lobe | Rare | Neg | Chronic Active | <25% (1+) | Few (1+) | Mixed active/inactive and demyelinating |
|  |  | CA2 |  | Rare | Very rare | Chronic Active | <25% (1+) | Few (1+) | Mixed active/inactive and demyelinating |
| 7 | B | 1 | Left frontal lobe | Rare | Rare | Chronic Active | <25% (1+) | Few (1+) | Mixed active/inactive and demyelinating |
| 8 | A | CA1 | Right frontal lobe | Rare | Rare | Chronic Active | <25% (1+) | Few (1+) | Mixed active/inactive and demyelinating |
|  |  | CA2 |  | Rare | Rare | Chronic Active | <25% (1+) | Few (1+) | Mixed active/inactive and demyelinating |
| 9 | A2 | 1 | Right inferior temporal lobe | Occ | Occ | Chronic Active | <25% (1+) | Few (1+) | Mixed active/inactive and demyelinating |
| 10 | B | CA1 | Right frontal lobe | Rare | Neg | Chronic Active | <25% (1+) | Few (1+) | Mixed active/inactive and demyelinating |
|  |  | CA2 |  | Rare | Rare | Chronic Active | <25% (1+) | Few (1+) | Mixed active/inactive and demyelinating |
| 11 | B6 | 1 | Left hippocampus and parahippocampal gyrus | Rare | Occ | Chronic Active | <25% (1+) | Few (1+) | Mixed active/inactive and demyelinating |
|  | | | | | | | | | |
| **Active Lesions** | | | | | | | | | |
| 1 | A3 | 1 | Left frontal lobe | NA | NA | NA | NA | NA | NA |
| 5 | A8 | 1 | Left parietal lobe | Pos | Pos | Active |  |  | Active and early demyelinating |
| 6 | E | 1 | Right posterior frontal lobe | Pos | Pos | Active |  |  | Active and early demyelinating |
| 8 | A | Active 1 | Right frontal lobe | NA | NA | NA | NA | NA | NA |
|  |  | Active 2 |  | Pos | Rare | Active |  |  | Active and early demyelinating |
|  | | | | | | | | | |
| **Controls** | | | | | | | | | |
| 1 | D | 1 | Right superior and middle temporal gyri |  |  |  |  |  |  |
| 2 | L | 1 | Right rostral cingulate gyrus |  |  |  |  |  |  |
| 3 | D | 1 | Left rostral middle frontal gyrus |  |  |  |  |  |  |
|  | G | 1 | Right inferior parietal lobe |  |  |  |  |  |  |
| 4 | H | 1 | Right rostral cingulate gyrus |  |  |  |  |  |  |

**Supplementary Table 3.**

Lesion classification scheme. Anon = anonymized, MƟ = Macrophages, Degree Demy = Degree of demyelination and score (*Kuhlmann et al, 2017*), Hyper cell border= Hypercellular border estimate and score (*Kuhlmann et al, 2017*), Pos = Positive (frequent positive cells in lesion), Rare= 1-2 positive cells in lesion border, Occ = Occasional (3-4) positive cells in lesion border. NA=tissue no longer available for additional classification.
